# The psychosocial impact of microtia and ear reconstruction: A national data-linkage study

**DOI:** 10.3389/fped.2023.1148975

**Published:** 2023-04-18

**Authors:** Thomas H. Jovic, John A. G. Gibson, Matthew Jovic, Thomas D. Dobbs, Rowena Griffiths, Ashley Akbari, Iain S. Whitaker

**Affiliations:** ^1^ Reconstructive Surgery and Regenerative Medicine Research Centre, Swansea University, Swansea, United Kingdom; ^2^ Welsh Centre for Burns and Plastic Surgery, Morriston Hospital, Swansea, United Kingdom; ^3^ Health Data Research UK, Swansea University, Swansea, United Kingdom

**Keywords:** microtia, depression, anxiety, education, data science

## Abstract

**Introduction:**

Children with visible facial differences are believed to be at increased risk of negative psychosocial behaviours which may manifest as affective disorders. The aim of this study was to determine whether a diagnosis of microtia, and the associated surgical intervention, is associated with psychosocial implications including impaired educational attainment and a diagnosis of an affective disorder.

**Methods:**

A retrospective case-control study was conducted using data linkage to identify patients in Wales with a diagnosis of microtia. Matched controls were sought on the basis of age, gender and socioeconomic deprivation status to yield a total sample size of 709. incidence was calculated using annual and geographic birth rates. Surgical operation codes were used to classify patients into those that had no surgery, autologous reconstruction or prosthetic reconstruction. Educational attainment at 11 years of age, plus a diagnosis of depression or anxiety were used as markers of adverse psychosocial outcomes and the relative risk was attained using logistic regression analyses.

**Results:**

There were no significant associations between a diagnosis of microtia and an increased risk of adverse educational attainment or a risk of an affective disorder diagnosis. Male gender and higher deprivation scores were significantly associated with poorer educational attainment, irrespective of a diagnosis of microtia. Surgical intervention of any nature was also not associated with any increased risk of adverse educational or psychosocial outcomes in microtia patients.

**Discussion:**

Microtia patients in Wales do not appear to be at greater risk of developing affective disorders or impaired academic performance as a result of their diagnosis or associated surgical intervention. Whilst reassuring, the need for appropriate support mechanisms to maintain positive psychosocial wellbeing and academic achievement in this patient cohort is reinforced.

## 1. Introduction

The presence of facial visible differences are associated with negative psychosocial behaviours that may manifest as reduced self-confidence, poorer quality of life and an increased risk of affective disorders (1–3). These behaviours are postulated to stem from a product of the diagnosis, perceptions of body image, and secondary to potential delays in the development of speech, language and hearing. Identifying affective disorders in children may be challenging: children may not experience psychosocial symptoms to the degree that clinical psychiatric diagnostic thresholds are met (4). Instead, issues such as social disintegration, low self-esteem, learning difficulties, poor school performance and overt anxiety and depression may be manifestations of the psychosocial difficulties faced by children with craniofacial anomalies (5–7). These behaviours may also manifest as bullying and social exclusion or poor academic performance and behavioural problems (8–10) which present psychosocial implications for both the patient (11) and their carers (12). The presence of craniofacial disorders in children aged 8–10 has been shown to be associated with higher rates of depression, anxiety and peer difficulties (such as teasing and bullying) than those aged 11–17 (13). Studies of patients with cleft lip and palate showed 59% had experienced bullying aged 8–11 compared to only 9% aged 4%–7% and 37% in children aged 12–15 (14).

Psychosocial issues have been particularly well described in the context of auricular anomalies such as prominent ears and microtia (15, 16). As with other craniofacial anomalies, similar age-related patterns have been observed in children with microtia, in which the 8–10 year age group and being of male gender were highlighted as significant risks for aggression and social problems, with female patients over the age of 17 being at greater risk of depression (11).

**Figure 1:**
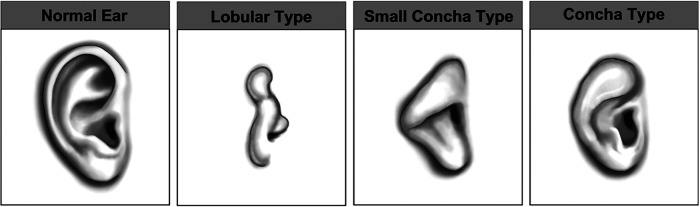
Nagata classification of auricular anomalies. Images kindly provided by Steve Atherton, Medical Illustration Department, Morriston Hospital, Swansea.

Microtia is a term used to encompass a spectrum of auricular anomalies affecting approximately 2–3 in every 10,000 live births in the United Kingdom (17) but with significant global variation in incidence (18). At its most severe, a complete absence of the auricle may occur (anotia), whereas milder malformations include conchal and lobular constrictions, which have be classified by Nagata (Figure 1) (19).

There has been evidence to suggest that support groups can have positive effects on psychosocial integration (20), and that surgical intervention is associated with improved psychosocial functioning and a reduction in manifestations of affective disorders in patients with microtia (21). A higher age of surgical intervention was associated with higher rates of depression and anxiety in this study, indicating the importance of timely surgical intervention to protect psychosocial wellbeing in these patients (21).

Current corrective options for microtia patients include autologous reconstruction with costochondral cartilage grafts, alloplastic reconstruction with silicone implants (e.g., Medpor) and bone anchored prostheses (22). Typically, autologous reconstruction is performed when the patient is between 8 and 10 years of age. Surgeons must balance the desire to intervene early to minimise disruption to psychosocial development with the need to delay untiul the auricle has reached approximately adult size and sufficient donor cartilage (23).

We have previously identified a cohort of microtia patients in Wales using data linkage, in which we discovered the incidence of microtia was almost double that previously reported (17, 18). Of the patients that underwent reconstructive surgery, we found that 72.9% opted for autologous reconstruction with the remainder receiving prosthetic ears (17). Using this cohort of patients, we sought to apply data linkage to determine whether a diagnosis of microtia was associated with adverse psychosocial functioning and whether the age or nature of surgical intervention was associated with any differences in risk of adverse psychosocial integration.

## 2. Methods

### 2.1. Study design

This retrospective, matched cohort study was designed and reported in accordance with the Reporting of studies Conducted using Observational Routinely-collected health Data (RECORD) statement (24).

### 2.2. Overview

Analysis of routine population-scale data from primary and secondary care National Health Service (NHS) and national administrative data sources for 2000–2018 in Wales, United Kingdom (population 3.1 million) was performed. In instances where relevant data were unavailable from a single source, multiple data sources were linked. Data was retrieved from five national data sources (Table 1). In Wales, population level de-identified person-based health and socio-economic administrative data are collated and linked within the Secure Anonymised Information Linkage (SAIL) Databank (25–27). Robust policies, structures and controls are in place to protect privacy through a reliable matching and anonymization process, achieved in conjunction with the NHS Wales Informatics Service (NWIS) using a split file multiple encryption approach (26).

**Table 1:** List of databases used for data linkage and their descriptions.

Database	Description
Annual District Death Extract (ADDE)	Collected from the Office for National Statistics (ONS), this database contains death registration information, relating to Welsh Residents including those who died outside of Wales.
Outpatient Dataset for Wales (OPDW)	Administrative and clinical data obtained from outpatient appointments in Wales.
Patient Episode Database for Wales (PEDW)	Administrative and clinical data for all hospital admissions, including diagnosis and operations performed.
Welsh Cancer Intelligence and Surveillance Unit (WCISU)	The national cancer registry for Wales. Captures all welsh melanoma patients from a number of sources; Multi-Disciplinary Team data, pathology data, other routine data sources in Wales and the English cancer registry.
Welsh Longitudinal General Practice dataset (WLGP)	Administrative and clinical data from all patient visits to a General Practitioner.
Welsh Demographic Service (WDS)	Administrative data about individuals resident or registered in Wales that have used National Health Service (NHS) services.

### 2.3. Study population

Microtia patients were identified from three separate sources, the Welsh Longitudinal General Practice (WLGP) data using READ codes for microtia and secondary care data obtained from Patient Episode Database for Wales (PEDW) and Outpatient Dataset for Wales (OPDW) using the International Classification of Disease 10 (ICD-10) code Q17.2 Microtia.

### 2.4. Matched controls

Controls were identified from the Welsh Demographic Service Dataset (WDSD). They were matched to the microtia cohort based on the following demographic variables: deprivation, sex and date of birth. Ten controls were sought for each case in the study. Deprivation was measured using the Welsh Index of Multiple Deprivation (WIMD) version 2011, a measure based on the Index of Multiple Deprivation and used as the official measure of socioeconomic status by the Welsh Government. Patients are assigned to one of five quintiles based on their postal code, with quintile 1 being the lowest socioeconomic status and 5 being the highest.

### 2.5. Study outcomes

#### 2.5.1. Operative procedures

Patient Episode Database for Wales (PEDW) was used to identify all day case and hospital admissions with inpatient surgical procedures recorded. Patients were classified as receiving no auricular reconstructive surgery, prosthetic reconstructive surgery or autologous reconstructive surgery based on appropriate Office of Population Censuses and Surveys Classification of Interventions and Procedures (OPCS-4) codes.

#### 2.5.2. School performance

Key stage 2 performance was used as an objective measure of school performance. In Wales, prior to the introduction of a new curriculum in September 2022, it was mandatory for schools to assess the performance of all learners aged 10 or 11 in their final year of KS2. Elsewhere in the United Kingdom, Key Stage 2 SATs are compulsory examinations sat between the ages of 10 and 11, however in Wales, these grades are made on the basis of teacher assessments. Performance in Mathematics, English and Science were extracted as levels ranging from 2 (the lowest score) to 6 (highest score).

#### 2.5.3. Risk of developing anxiety disorders and depression

A diagnosis of an anxiety disorder or depression was established from the Welsh Longitudinal General Practice (WLGP) data, as recorded during consultations with patients in General Practitioner (GP) records, using Read codes that have been previously validated As the diagnoses of anxiety and depression were based on GP records, patients not enrolled with a general practice that contributes data to the SAIL Databank were excluded. SAIL holds data on approximately 80% of general practices around Wales.

### 2.6. Ethical approval

Approval for the use of anonymised data in this study, provisioned within the Secure Anonymised Information Linkage (SAIL) Databank was granted by an independent Information Governance Review Panel (IGRP) under project 0651. The IGRP has a membership comprised of senior representatives from the British Medical Association (BMA), the National Research Ethics Service (NRES), Public Health Wales and NHS Wales Informatics Service (NWIS). Usage of additional data was granted by data owner. The SAIL Databank is General Data Protection Regulations (GDPR) and the UK Data Protection Act compliant.

### 2.7. Statistical analysis

Descriptive statistics were used to characterise the microtia cases, and a chi squared test used to determine statistically significant disparities between the cohorts.

In order to observe how microtia (along with the other variables in our data set) affects school performance, an ordinal regression (Proportional Odds Model) was undertaken. A diagnosis of microtia, age, gender, socioeconomic deprivation and nature of surgical intervention were used as covariates A test of parallel lines checked the condition of proportional odds, which all ordinal regressions resulted in a *p*-value > 0.05, verifying the assumption of proportional odds.

Logistic regression analyses were performed to determine the association between a diagnosis of anxiety disorders and depression with microtia. A diagnosis of microtia, age, gender, socioeconomic deprivation and nature of surgical intervention were used as covariates.

A diagnosis of microtia, age, gender, socioeconomic deprivation and nature of surgical intervention were used as covariates in a logistic regression analysis to determine the odds ratio of anxiety or depression and in an ordinal regression analysis to predict KS2 performance. Using Variance Inflation Factors it was identified that microtia and operations demonstrated collinearity and as such were assessed using separate regression models. All data were analysed using IBM SPSS Statistics for Windows (IBM Corp. Released 2017. Version 25.0. Armonk, NY: IBM Corp). Statistical significance was assumed with a *p* < 0.05.

## 3. Results

### 3.1. Demographics

A total of 72 microtia cases were identified above the age of 11, of which 63% were male. The median age of patients in this cohort was 21 with a median deprivation score of 2.637. Age and gender matched controls were identified to give approximately a 1:9 case:control ratio and a total study number of 709. Demographically, there were no statistically significant differences in the age (*p* = 0.960), sex (*p* = 0.857) or WIMD quintile (*p* = 0.239) between case and control cohorts (Table 2).

**Table 2:** Demographics of case and control populations at the time of analysis (Jan 2022).

	Control (*n* = 637)	Microtia Cases (*n* = 72)	*p* value
**Age (at time of analysis), *n* (%)**			
11–15	72 (11)	7 (10)	0.960
16–20	230 (36)	26 (36)	
21–25	258 (41)	31 (43)	
26–30	77 (12)	8 (11)	
Median (IQR)	19 (16–22)	21 (18–24)	
**Sex, *n* (%)**			
Male	405 (64)	45 (63)	0.857
Female	232 (36)	27 (37)	
**WIMD Quintile, *n* (%)**			
1	129 (20)	23 (32)	0.239
2	109 (17)	8 (11)	
3	88 (14)	8 (11)	
4	82 (13)	10 (14)	
5	59 (9)	7 (10)	
Unspecified	170 (27)	16 (22)	
Median (IQR)	2 (1–4)	2 (1–4)	
Key stage 2 results			
**English**			
2	8 (1)	0 (0)	0.558
3	85 (13)	<10	
4	289 (45)	38 (53)	
5	241 (38)	25 (35)	
6	8 (1)	0 (0)	
Unspecified	6 (1)	<10	
Median (IQR)	4 (4–5)	4 (4–5)	
**Maths**			
2	12 (2)	0 (0)	0.388
3	74 (12)	<10	
4	306 (48)	39 (54)	
5	237 (37)	23 (32)	
6	<10	<10	
Unspecified	<10	<10	
Median (IQR)	4 (4–5)	4 (4–5)	
**Science**			
2	6 (1)	0 (0)	0.603
3	71 (11)	<15	
4	327 (51)	34 (47)	
5	227 (36)	26 (36)	
Unspecified	6 (1)	<15	
Median (IQR)	4 (4–5)	4 (4–5)	
**Depression**			
Diagnosed	49 (8)	5 (7)	
**Anxiety**			
Diagnosed	43 (7)	<5 (<7)	

Regarding academic performance, there were no statistically significant disparities detected between the case and control populations for English (*p* = 0.558), Mathematics (*p* = 0.388) or Science (*p* = 0.603). There were also no statistically significant disparities in the incidence of anxiety (*p* = 0.399) or depression (*p* = 0.655) in either cohort.

### 3.2. The association between school performance and microtia

Ordinal regression analysis results are displayed in Table 3. A diagnosis of microtia was not associated with lower school performance across Maths, English or Science. Moreover, the nature of surgical intervention was not associated with school performance.

**Table 3:** Summary table of regression analyses to determine odds ratios of age, depression anxiety, microtia, gender, deprivation and surgical intervention on KS2 assessment performance.

Ordinal Regression	English		Maths		Science	
Variable	*p* value	Odds (CI)	*p* value	Odds (CI)	*p* value	Odds (CI)
Age	**0** **.** **01**	**0.93** (0.88–0.98)	0.09	0.95 (0.90–1.01)	0.50	0.98 (0.93–1.04)
Depression	0.65	0.87 (0.48–1.58)	0.91	0.97 (0.53–1.76)	0.83	1.07 (0.58–1.96)
Anxiety	0.70	0.88 (0.46–1.68)	0.98	1.01 (0.53–1.93)	0.66	0.86 (0.45–1.66)
Microtia	0.89	0.96 (0.56–1.64)	0.67	1.12 (0.66–1.93)	0.73	1.10 (0.64–1.90)
Gender (male)	**0** **.** **02**	0.65 (0.45–0.92)	0.64	0.92 (0.64–1.31)	0.07	0.71 (0.50–1.02)
WIMD						
1	**<0** **.** **001**	**0.31** (0.18–0.56)	**<0** **.** **001**	**0.31** (0.17–0.54)	**<0** **.** **001**	**0.20** (0.11–0.36)
2	**0** **.** **02**	**0.50** (0.28–0.90)	**<0** **.** **001**	**0.32** (0.18–0.59)	**<0** **.** **001**	**0.29** (0.16–0.54)
3	0.07	0.56 (0.31–1.05)	**0** **.** **01**	**0.45** (0.24–0.84)	**0** **.** **003**	**0.38** (0.20–0.72)
4	0.08	0.58 (0.31–1.07)	**0** **.** **007**	**0.42** (0.22–0.78)	**0** **.** **013**	**0.45** (0.24–0.85)
5	-	-	-	-	-	-
Prosthetic	0.95	0.97 (0.49–1.98)	0.86	1.06 (0.52–2.13)	0.67	1.17 (0.58–2.36)
AUtologous	0.95	1.03 (0.37–2.85)	0.81	0.88 (0.31–2.45)	0.81	1.14 (0.40–3.20)

Statistically significant results are depicted in bold.

There was noted to be a significant association between English assessments with age (at time of analysis) and gender. An increased age (at time of analysis) showed a decrease in the odds of scoring a higher English assessment score, with an odds ratio of 0.93 [Wald *χ*^2^(1) = 6.378, *p*-value = 0.012] and that males had a lower likelihood of high assessment scores than females with an odds ratio of 0.65 [Wald *χ*^2^(1) = 5.756, *p*-value = 0.016]. This indicates that performance in English assessments has improved in more recent years but that female students perform significantly better than males, irrespective of a diagnosis of microtia.

Socioeconomic deprivation was a predictor of assessments performance in each KS2 subject, with individuals in WIMD quintiles 1 and 2 having a significantly reduced odds of attaining higher level assessments results compared to those in the 5th WIMD quintile. This indicates that those from lower socioeconomic backgrounds are at a more likely to achieve lower KS2 results than those in higher socioeconomic brackets.

### 3.3. The association of anxiety disorders and depression with microtia

Anxiety disorders were not found to be significantly associated with a diagnosis of microtia (OR 0.60 95% CI 0.18–1.99, *p* = 0.4) using logistic regression analyses (Table 4). Microtia patients who underwent autologous reconstructive surgery did appear to have higher odds ratios of developing anxiety at 1.73 (0.15–19.98, *p* = 0.66) but this was not statistically significant and presented with a large confidence interval, likely reflecting the small sample size.

**Table 4:** Logistic regression analysis of microtia and affective disorders.

	*p* value	Odds	95% CI	
**Anxiety**				
Microtia	0.404	0.601	0.182	1.987
Reconstruction	0.661	1.73	0.15	19.978
**Depression**				
Microtia	0.821	0.896	0.345	2.325
Reconstruction	0.787	1.292	0.203	8.236

Similarly for depression, there was no association with a diagnosis of microtia and an increased odds of a diagnosis of depression. As with anxiety, autologous reconstructive surgery was associated with a mildly elevated odds of depression (1.29) but this was not statistically significant (*p* = 0.787) and with a wide confidence interval, meaning no associations can be concluded.

### 3.4. The effects of surgical intervention on school performance and affective disorders

Of the patients that had a diagnosis of microtia, 39 (54%) were known to have undergone ear reconstruction surgery whereas 33 (46%) had no documented ear reconstruction surgery. In this cohort, 13% of these patients were coded as having prosthetic ears inserted whereas 87% had plastic surgery operations on the ear ranging from minor corrective surgery to total auricular reconstruction. Of the patients with microtia, there was no statistically significant difference in the KS2 assessment results of those patients having surgical intervention compared to those who did not have a recorded surgical intervention (Table 5). Anxiety and depression were uncommon in both the surgical and non-surgical cohorts but whether or not microtia patients had operative intervention did not appear to convey any significant risk.

**Table 5:** Demographics of patients with microtia (operative intervention and no operative intervention), and associations with affective disorders and key stage 2 assessments performance.

	Operation (*n* = 39)	No operation (*n* = 33)	*p*-value
**Age (at time of analysis), *n* (%)**			0.2133
11–15	<16	<12	
16–20	<16	<12	
21–25	18 (46)	13 (39)	
26–30	<16	<12	
Median (IQR)	21 (18–24)	21 (18–23)	
**Gender, *n* (%)**			1
Male	24 (62)	21 (64)	
Female	15 (38)	12 (36)	
**WIMD, *n* (%)**			0.2414
1	13 (33)	10 (30)	
2	6 (15)	<10	
3	<10	<10	
4	<10	5 (15)	
5	<10	<10	
Unspecified	7 (18)	<10	
Median (IQR)	2 (1–4)	2.5 (1–4)	
**KS2**			
**English, *n* (%)**			0.1991
2	0	0	
3	5 (13)	<15	
4	21 (54)	17 (52)	
5	13 (33)	<15	
6	0	0	
Unspecified	0	0	
Median (IQR)	4 (4–5)	4 (4–5)	
**Maths, *n* (%)**			0.2133
2	0	0	
3	5 (13)	<11	
4	21 (54)	18 (55)	
5	13 (33)	<11	
6	0	<11	
Unspecified	0	0	
Median (IQR)	4 (4–5)	4 (4–5)	
**Science, *n* (%)**			0.1991
2	0	0	
3	8 (21)	<15	
4	18 (46)	16 (48)	
5	13 (33)	<15	
6	0	0	
Unspecified	0	0	
Median (IQR)	4 (4–5)	4 (4–5)	
**Depression**			
Diagnosed	<5	<5	
**Anxiety**			
Diagnosed	<5	<5	

## 4. Discussion

This study aimed to apply data linkage methodology to assess the impact of microtia and its associated surgical management on the psychosocial wellbeing of patients.

There have been a number of previous research articles that have identified associations between psychosocial problems and children with craniofacial anomalies. Whilst a significant proportion of these have focussed on cleft lip and palate patients (5, 7, 20, 28, 29), there are a number that have focussed primarily on microtia (11, 15, 21), and recurrent themes such as diagnostic stigma, perceptions of body image, and delays in the development of speech, language and hearing may underpin the profound effects these diagnoses can have on patients and their families. Indeed, stress, anxiety and depression have all been noted in patients as young as pre-school ages in parental studies of craniofacial microsomia (30). Specifically, previous studies of microtia patients have identified that patients who do not have reconstructive surgery are at greater risk of developing issues such as depression, hostility, aggression and social difficulties (15). Earlier surgical intervention is believed to negate these risks, and studies have demonstrated that surgical intervention alleviates some patient and parent reported negative emotions and improves social awareness (21). In expert hands, autologous ear reconstruction can offer excellent results for patients, and high levels of post-operative patient reported satisfaction have been described (31–33). However in this study, we were unable to confirm whether or not surgical intervention was protective against affective disorders as the incidence of anxiety and depression was so low in this cohort.

A limitation to the data acquired in this field to date has been the subjective nature of its acquisition through questionnaire-based methods. Whilst these measures are an essential means of exploring the patient's perspective and assessing the psychosocial impact of microtia they are subject to a number of biases and subjectivity (34). Data linkage has the power of analysing the association between objective measures of academic attainment: in this case KS2 assessment results, and formal diagnoses of anxiety disorders and depression sustained throughout life. The absence of a positive association in this case is a reassuring finding, as this could indicate that sufficient psychological support is available for these patients in the context of their home and school environment to allow for psychosocial integration and normal academic attainment: a testament to those involved in the multidisciplinary care of these patients. However, some caution should be exercised in interpreting these findings, as the number of cases of microtia are relatively small, and those with affective disorder diagnoses are smaller still, meaning an absence of statistical significance may be masking a clinically significant finding. Additionally, there may be underreporting of the incidence of affective disorders in this cohort owing firstly to inaccuracies in clinical coding, and secondarily attributable to the diagnostic challenges and stigma associated with diagnosing affective disorders in early stages of life (35, 36). From our own anecdotal experience, we anticipated the proportion of prosthetic ears to be higher than 17% of reconstructions, which may reflect inaccuracies or ambiguity in coding. Furthermore, whilst data linkage is a powerful means of detecting diagnostic information across a range of health and social databases, the anonymisation hinders acquisition of additional information about the severity of the auricular anomaly and the extent of surgical intervention required. This limitation could also mask significant associations in patients with more severe auricular anomalies or needing more extensive surgical intervention.

Children with microtia in this cohort achieved comparable school results to age matched controls which is a highly relevant finding in light of the potential effects of microtia on hearing, self-confidence and absence from school to attend outpatient appointments and surgical inpatient stays. It has been previously reported that patients with craniofacial microsomia may be disadvantaged in academic settings owing to restricted participation (37), however comparable academic attainment was achieved in Maths, Science and English in our cohort at 10–11 years of age. Further validation of this finding could be attained through an analysis of GCSE and A-level data to ascertain whether any latent effects on academic attainment manifest later in life. Nevertheless, it is reassuring that academic attainment is preserved at the pivotal stage of KS2, around the age of surgical intervention and the emergence of increased psychosocial awareness.

## 5. Conclusion

In this study we have identified that microtia patients in Wales do not appear to be at greater risk of developing affective disorders or impaired academic performance as a result of their diagnosis or associated surgical intervention. Whilst reassuring, further work should seek to clarify whether longstanding effects manifest with regard to educational attainment and whether disease severity and the extensiveness of surgery are associated with adverse psychosocial integration in larger cohorts. In the interim, the need for identifying appropriate support mechanisms to maintain positive psychosocial wellbeing and academic achievement in this patient cohort is reinforced.

## Data Availability

The original contributions presented in the study are included in the article/Supplementary Material, further inquiries can be directed to the corresponding author.
